# Characterization and structure of hypomania in a British nonclinical adolescent sample

**DOI:** 10.1016/j.jad.2016.08.033

**Published:** 2017-01-01

**Authors:** Georgina M. Hosang, Alastair G. Cardno, Daniel Freeman, Angelica Ronald

**Affiliations:** a Psychology Department, Goldsmiths, University of London, London SE14 6NW, UK; b Academic Unit of Psychiatry and Behavioural Sciences, University of Leeds, Leeds Institute of Health Sciences, Charles Thackrah Building, University of Leeds, 101 Clarendon Road, Leeds LS2 9LJ, UK; c Department of Psychiatry, University of Oxford, Warneford Hospital, Oxford OX3 7JX, UK; d Department of Psychological Sciences, Birkbeck, University of London, Malet Street, London WC1E 7HX, UK

**Keywords:** Hypomania, Hypomanic Checklist, HCL, Bipolar disorder, Adolescence, Youth

## Abstract

**Background:**

This study aimed to test the validity of using the Hypomania Checklist-16 [HCL-16] to measure hypomania in a British adolescent community sample. Limited research is available concerning the characterization of hypomania among community adolescent samples, particularly in the UK, despite its potential importance for early intervention policy development.

**Method:**

To explore the structure and characterization of hypomania in a British adolescent nonclinical cohort, over 1400 17 year olds (Mean=17.05 years; SD=0.88) completed the HCL-16 along with measures of different psychological and psychopathological dimensions.

**Results:**

Principal components analysis revealed a 2-component solution for the HCL-16, described as active-elated and irritable/risk-taking. Hypomanic symptoms were significantly correlated with many psychopathological dimensions. There were distinct correlation patterns for the two HCL-16 subscales, with the irritability/risk-taking subscale showing significantly stronger associations with psychotic-like experiences, internalizing and externalizing problems, and reduced life satisfaction relative to the active-elated dimension. Adolescents at ‘high-risk’ for bipolar disorder reported more psychopathology relative to the comparison group.

**Limitations:**

Absence of the clinical diagnosis of bipolar disorder in the sample means that the classification of the ‘high-risk’ group cannot be confirmed.

**Conclusions:**

The structure of the HCL-16 in this UK adolescent sample mirrored that observed in adult and clinical cohorts. The observed links between the HCL-16 and psychopathological dimensions that have been previously associated with both hypomania and bipolar disorder lend support to the HCL-16's validity as a hypomania instrument for adolescents. Better understanding of hypomania prior to adulthood has considerable potential for informing early intervention approaches.

## Highlights

• HCL-16 items mapped onto two components: active-elated and irritable/risk-taking.
• The HCL-16 score was associated with psychopathological and psychological problems.
• The correlation patterns for the two HCL-16 subscales were different.
• Irritable/risk-taking showed the strongest links to psychopathological symptoms.

## 1 Introduction

The average age of onset for bipolar disorder [BD] ranges from 18 to 22 years (Merikangas et al., 2011), although up to 60% of people with BD report their illness onset occurring in childhood or adolescence (Perlis et al., 2009). There has been a rise in the clinical diagnosis of pediatric BD (Moreno et al., 2007), with a meta-analysis reporting a prevalence of 1.8% in community samples (Van Meter et al., 2011). Thus the true age of onset may occur earlier than what is documented. Subsyndromal hypomanic symptoms in youth have been linked to similar severity and impairment experienced by those with clinically diagnosed BD and have been associated with subsequent clinical hypomanic or manic episodes (Axelson et al., 2015). Therefore a better understanding of the characterization of hypomania during adolescence may help improve accurate and timely diagnosis of BD. Furthermore, there is increased interest surrounding pediatric BD, but there is limited research in the UK with the majority of the published work conducted in the US (Skirrow et al., 2012). This means it is unclear whether the findings from US samples are generalizable to the UK and other populations.

Currently, identification of young people at ‘high-risk’ of developing BD (particularly in community samples) involves screening for the presence of hypomanic symptoms (Waugh et al., 2014). Another approach is to focus on other elements of the diagnostic criteria, such as the impact on functioning and symptom duration (Meyer et al., 2007). Using German and Swedish community samples, researchers have found that those categorized as ‘high-risk’ adopting this approach report experiencing externalizing (e.g. hyperactivity/inattention) and internalizing (e.g. anxiety symptoms) problems (Holtmann et al., 2009), which mirror the common comorbidities observed in BD (Merikangas et al., 2011). Although these findings provide support for the validity of this approach of identifying individuals at ‘high-risk’ of developing BD, such problems are fairly common in youth (Goodman et al., 2000). Examination of whether other psychopathological and psychological dimensions known to co-occur with BD are also observed among ‘high-risk’ individuals, such as psychotic-like experiences (eg. paranoia) is needed. Also it would be of value if a more comprehensive method of identifying young people at risk of developing BD was developed which combines existing screening methods and which reflects all the key elements of diagnosis (i.e. the presence of symptoms, their impact and duration), especially given the challenges of diagnosing pediatric BD (Youngstrom et al., 2009). This study will explore the validity of this more comprehensive method of identifying adolescents that show vulnerability to BD, which could have implications for clinical practice.

Mania and hypomania can be divided into two sides or dimensions: the ‘bright’ and ‘dark’ (Hantouche et al., 2003). The ‘bright’ side is concerned with the socially advantageous aspects of hypomania (eg. elation), and is also referred to as active-elated (Brand et al., 2011) or exuberant (Stringaris et al., 2011). The ‘dark’ side is related to the socially negative facets of hypomania (eg. irritability) and is also referred to as irritability/risk-taking (Brand et al., 2011) or under-controlled (Stringaris et al., 2011). Research shows that these two sides of hypomania are differentially related to psychological impairment and comorbid symptomatology. For example, the ‘dark’ side of hypomania has been more strongly related to symptoms of depression, sleep problems and less life satisfaction compared to the ‘bright’ side (Brand et al., 2011), whereas the ‘bright’ side is associated with higher verbal IQ (Stringaris et al., 2014).

The overall aim of this study was to test the validity of using the Hypomania Checklist-16 [HCL-16] (Forty et al., 2010) to measure hypomania in a British adolescent community sample. This aim was achieved through the investigation of four specific objectives. First, the component structure of hypomania in a nonclinical sample of British adolescents was explored. The second objective was to assess the construct validity of the HCL-16 through the assessment of the extent to which the HCL-16 scores were associated with other dimensions of psychopathology that are known to be related to clinical levels of hypomania and BD. These included psychotic-like experiences as well as internalizing (e.g. depression) and externalizing (e.g. hyperactivity/ inattention) problems. In addition, the relationships between hypomania, personality, life satisfaction and sleep problems were investigated. Exploration of these relationships was undertaken since there is evidence of links between sleep disturbance and reduced life satisfaction with clinical levels of hypomania (Harvey, 2008, Michalak et al., 2005). Previous research also shows that BD is associated with extraversion, openness to experience, and neuroticism (Tackett et al., 2008). Third, differences between the two hypomania dimensions (‘bright’: active-elated and ‘dark’: irritable/risk-taking) were examined in terms of their psychological and psychopathological correlates. The final objective was to explore whether dimensions of the hypomania measure could identify, using cross-sectional data, a group of adolescents that show signs of vulnerability to BD.

## 2 Method

### 2.1 Participants

This study consisted of 1440 adolescent individuals, 58% (N=830) females, with a mean age of 17.05 years (SD=0.88; range: 15–19 years) who took part in the second phase of the Longitudinal Experiences And Perceptions [LEAP] project (Ronald et al., 2014). Some of the data analysed in this study was originally collected at the first phase of the LEAP study, which occurred on average 9 months earlier. For more details of the sample used in the present study see Table 1. The LEAP sample consists of twin pairs and their parents drawn from the Twins Early Development Study (Haworth et al., 2013). For the purposes of the current investigation only one twin from each pair was included. LEAP received full ethical approval and informed consent was provided by all participants, for further details on the study see (Ronald et al., 2014).

### 2.2 Materials

Lifetime hypomanic symptoms were measured using the self-report Hypomania Checklist-16 [HCL-16] (Forty et al., 2010). The HCL-16 instructs participants to recall a period at any point in their lives when they were in a ‘high’ state or their mood was more ‘up’ than usual. ‘Highs’ were operationalized as increases in energy, activity and mood. Participants then indicate the presence of 16 behaviours, thoughts and emotions related to hypomania during these ‘high’ phases using statements such as ‘I need less sleep’. A yes/no response is given for each statement, providing a minimum score of 0 and a maximum score of 16 for the instrument. In the current study one item was removed (‘I feel more flirtatious and/or am more sexually active’) to avoid offending participants of this longitudinal study.

The HCL-16 also includes items enquiring about the duration and impairment of the ‘high’ periods (overall not individual symptoms). Participants recorded the duration of the ‘high’ periods using six options ranging from ‘1 day′ to ‘longer than a month’. The specific impact of the ‘high’ phases on four life domains (ie. social, family, leisure and school/work/college) was rated using four option: no impact, negative, positive, negative and positive. Participants also reported on other people's reactions to their ‘high’ periods using five responses: positively, neutral, negatively, positively and negatively and no reactions.

The HCL-16 is based on the longer HCL-32 version originally designed to distinguish between major depression and BD in clinical samples (Forty et al., 2010). Both versions have been shown to have good psychometric properties in adults (Angst et al., 2005, Forty et al., 2010) and have been used previously in adolescent cohorts (Holtmann et al., 2009). For instance, the HCL-32 has good specificity (0.79) and sensitivity (0.85) in distinguishing people with BD from healthy controls or those with major depression (Vieta et al., 2007). The HCL-16 was completed at phase 2 of the Leap Project.

### 2.3 Questionnaires completed at phase 2 of the Leap Study (including HCL-16)

Depressed mood over the past 2 weeks was measured using the Short version of the Moods and Feelings questionnaire [SMFQ] designed for children and adolescents (Angold et al., 1995). The 13 SMFQ items map onto the core symptoms of depression, which are rated with a yes/no response.

Anxiety sensitivity was assessed using the Child Anxiety Sensitivity Index (Silverman et al., 1991) which consists of 18 statements rated on the degree to which they are true for the participants in the last six months using a 3-point Likert scale ranging from ‘none’ to ‘a lot’.

Psychotic-like experiences were assessed using two instruments. The first instrument is the Specific Psychotic Experiences Questionnaire [SPEQ] which has been previously validated in this adolescent sample (Ronald et al., 2014) and assesses 6 types of psychotic-like experiences (timeframe enquired about provided in brackets): paranoia (current, no timeframe specified), hallucinations (current, no timeframe specified), cognitive disorganization (past month), grandiosity (past month), anhedonia (past month) and negative symptoms (current, no timeframe specified). Five of the six types of psychotic-like experiences are measured using self-report with negative symptoms based on parent report. The second instrument was the Psychotic-Like Symptoms Questionnaire [PLIKS-Q] (Zammit et al., 2011), which was originally developed using adolescents. This 11-item self-report instrument covers symptoms of hallucinations, delusions and thought interference experienced in the last year. Participants were asked to report if they had these experiences and if so, how frequent and distressing they were.

Family history of BD among first and second-degree relatives was provided by parent report.

Socioeconomic status (SES) composite score was based on parental education, occupation and family income. The score was created as an unit-weighted sum of the contributing measures (Hanscombe et al., 2012).

Substance use was assessed by asking participants whether they had used 15 different substances (eg. cannabis) in the past 12 months. ‘Yes’ responses were classified as positive for substance use.

### 2.4 Questionnaires completed at phase 1 of the Leap Study (9 months prior to the HCL-16)

Emotional and behavioural problems experienced in the last 6 months were measured using the Strengths and Difficulties Questionnaire [SDQ] designed for children and adolescents (Goodman, 1997). This self-report instrument consists of 25 items rated on a 3-point Likert scale ranging from ‘not true’ to ‘certainly true’. The SDQ is comprised of 5 subscales: emotional problems, prosocial behaviour, conduct problems, hyperactivity/inattention and peer problems.

Personality traits were assessed using a 30-item self-report questionnaire that maps onto the traditional 5-factor model validated and developed using adolescent samples (Mullins-Sweatt et al., 2006).

Life satisfaction was measured using the Brief Multidimensional Students Life Satisfaction Scale which asks respondents to rate how satisfied they are currently with five life domains (eg. family), with one item enquiring about overall life satisfaction (Seligson et al., 2003). Each domain is rated on a 7-point Likert scale ranging from ‘very dissatisfied’ to ‘very satisfied’. This instrument is designed to be completed by children and adolescents (Seligson et al., 2003).

Sleep quality over the last month was assessed using the Pittsburgh Sleep Quality Index (Buysse et al., 1989) which consists of 17 items which cover a range of dimensions and disturbances including sleep duration, difficulties falling and staying asleep. Scores ranged from 0 to 21, with higher scores indicative of poorer sleep quality. This instrument has been used previously in adolescent cohorts (Lund et al., 2010) and was completed at the first phase of the LEAP study (Taylor et al., 2015).

### 2.5 Analyses

One twin per pair was included in all analyses, randomly selected for birth order. Principal components analyses [PCA] were conducted to explore the structure of the HCL-16. PCA were undertaken using Varimax rotation and component extraction was based on visual analysis of the scree plot. Spearman's correlations were used to explore associations between hypomania (scores from the total scale and the subscales) and other dimensions. T-tests or χ^2^ tests were used to explore group differences; bonferroni corrections were applied when multiple tests were conducted. The significance of the group differences was assessed using Cohen's d, which measured the effect size, where 0.2 was considered small, 0.5 medium and 0.8 indicating a large effect. Steiger's Z tests were used to test any significant differences between two dependent correlations (eg. correlations between psychopathological dimensions and the two hypomania subscales) (Steiger, 1980). All data were analysed using version 22 of SPSS.

## 3 Results

The mean HCL-16 score for the entire sample was 7.12 (SD=2.60); no significant differences in the HCL-16 score between genders (t(1438)=0.82, p=0.41), and those from Caucasian and other ethnic backgrounds (t(1434)=1.16, p=0.25) were observed. A significant positive correlation was detected between SES and hypomanic symptoms (r_s_=0.15, p≤0.001). Those that reported using substances in the last 12 months recorded significantly more hypomanic symptoms compared to individuals that didn’t report such use (t(1433)=5.49, p≤0.001).

### 3.1 Psychometric properties of the HCL-16

Based on examination of the scree plot, 3 components were extracted which each had Eigenvalues of 2.72, 1.77 and 1.11 respectively; together the components explained 37.34% of the variance. The third component only consisted of 3 items (items 3, 4 and 8; listed in Table 2), with item 3 cross loading onto Component 1. Component 3 appeared quite heterogeneous given that the items appear to map onto expansive mood, risk-taking and increased energy. Furthermore, Component 3's Cronbach's alpha of 0.39 was poor.

To allow for comparisons with the 2-component models identified for the HCL (Angst et al., 2005), the principal component analysis was restricted to a 2-component solution. For Component 1 the Eigenvalue was 2.72 and explained 18.16% of the variance (Table 2). Nine items loaded onto this component (eg. item 1: need less sleep; item 6: more active), which closely resembled the ‘active-elated’ subscale (or ‘bright’ side of hypomania) established previously for the HCL in other samples. The Eigenvalue for Component 2 was 1.77 accounting for 11.81% of the variance. This component consisted of 6 items and was similar to the ‘irritable/risk-taking’ subscale (or ‘dark’ side of hypomania) previously identified for this questionnaire (Forty et al., 2010). Example items from this scale include spending more money (item 4) and having more quarrels (item 11). A total of 29.97% of the variance was explained by this 2-component model comparable with previous studies using this instrument (Forty et al., 2010). A moderate correlation between the two components from the principal component analysis was found (r=0.43, p=≤0.001) and the correlations between items ranged from r=0.002 and r=0.33.

The items most frequently endorsed in this sample were: higher mood/more optimistic, and less shy or inhibited (Table 2). The following items were endorsed the least: smoking more cigarettes and taking more drugs (Table 2). The Cronbach's alphas for the HCL-16 in this sample was 0.66; 0.67 for the first subscale (‘bright’ side or active-elated) and 0.42 for the second subscale (‘dark’ side or irritable/risk-taking).

### 3.2 Family history of BD

Individuals with a family history of BD had higher mean scores on the HCL-16 (t(1420)=1.37, p=0.17) and on the active-elated subscale (t(1395)=1.99, p=0.047) compared to those without such family history but only the latter was significant. Similar mean scores on the irritable/risk-taking scale were found among those with and without a family history of BD (t(1394)=0.67, p=0.50).

### 3.3 Correlations between HCL with psychological and psychopathological dimensions

The associations between HCL-16 (total score and its two subscales) and various psychological and psychopathological dimensions are presented in Table 3. The HCL-16 total score was significantly correlated with all of the psychotic-like symptoms, depressed mood and anxiety sensitivity, except for SPEQ Negative symptoms (r_s_=0.01, p>0.002). Other constructs were measured prior to the HCL-16 (SDQ and all of the remaining psychological dimensions), therefore the analyses considering the associations between these dimensions were undertaken in an exploratory capacity. These analyses revealed significant correlations between the HCL-16 total score with all of the SDQ subscales except for peer problems (r_s_=0.05, p>0.002) and prosocial behaviour (r_s_=0.06, p>0.002). The HCL-16 total score was also significantly related to sleep problems (r_s_=0.20, p≤0.002) and decreased life satisfaction (r_s_=−0.09, p≤0.002). Exploratory analyses only showed significant associations with two personality constructs: extraversion (r_s_=0.18, p≤0.002) and openness to experience (r_s_=0.14, p≤0.002).

There were distinct correlation patterns for the two HCL-16 subscales, with the ‘dark’ side or irritability/risk-taking subscale showing significantly stronger associations with all of the psychotic-like experience dimensions and depressed mood, with only a few exceptions. The ‘bright’ and ‘dark’ side of hypomania showed comparable correlations with SPEQ Grandiosity (z=0.26, p>0.002), SPEQ Hallucinations (z=2.07, p>0.002) and psychotic-like symptoms measured by the PLIKS-Q (z=2.68, p>0.002). SPEQ Anhedonia was more strongly correlated to the ‘bright’ rather than the ‘dark’ side of hypomania (z=2.14, p=0.032). In comparison, the exploratory analyses showed that the ‘dark’ side had significantly stronger correlations relative to the ‘bright side’ for 5 of the 12 dimensions. These were: SDQ emotional problems (z=3.79, p≤0.002), SDQ hyperactivity (z=5.30, p≤0.002), SDQ conduct problems (z=4.85, p≤0.002), SDQ peer problems (z=5.52, p≤0.002), and lower life satisfaction (z=−4.99, p≤0.002).

### 3.4 Profile of individuals who were hypothesized to be at ‘high-risk’ for BD

Individuals at ‘high-risk’ for BD were categorized as those with a HCL-16 score of 8 or more (instrument cut-off), whose highs lasted at least 2 days and were associated with negative consequences. These criteria take into account the key elements of the diagnostic criteria: number, impact/impairment and duration of symptoms (APA, 2013). Given the debate surrounding the possible shorter episode duration in pediatric BD (Stringaris et al., 2010), ‘high’ phases of 2 days or more was the focus here.

Based on the outlined criteria, 124 participants (9% of the sample) were categorized as ‘high-risk’ and 1316 individuals formed the comparison group. The ‘high-risk’ group reported significantly more symptoms of psychotic-like experiences (except for negative symptoms), depression and anxiety sensitivity relative to the comparison group with small effect sizes. The exploratory analyses revealed several differences with small effect sizes although the *t*-tests were non-significant. Specifically the ‘high-risk’ group's mean score on openness to experience (t(565)=2.78, p>0.002, d=0.44), neuroticism (t(567)=2.65, p>0.002, d=0.43), SDQ conduct problems (t(1434)=2.51, p>0.002, d=0.24) and SDQ emotional problems (t(1434)=1.92, p>0.002, d=0.28) were higher than the comparison group. For life satisfaction the ‘high-risk’ group's mean score was lower than that of the comparison group (t(1435)=2.64, p>0.002, d=0.26) (Table 4).

The DSM-5 diagnostic criteria for hypomania requires symptom duration of at least 4 days. Therefore among the ‘high-risk’ group (reporting 8 or more hypomanic symptoms which were associated with negative consequences), those with briefer ‘high’ phases (2–3 days) were compared to those with ‘highs’ that lasted longer (4 or more days) on the psychopathological and psychological dimensions. No significant differences were observed between these groups (supplementary Table 1).

## 4 Discussion

This study investigated the structure and characterization of hypomania in a large British adolescent sample. This study's four specific objectives will be explored in turn in the following sections.

### 4.1 Psychometric properties of the HCL

The first objective of this study was to explore the structure of hypomania in a nonclinical sample of British adolescents. The findings of this study showed that the HCL items map onto two components: active-elated and irritable/risk-taking, also referred to as the ‘bright’ and ‘dark’ sides of hypomania respectively. These results are comparable to previous studies using clinical (Forty et al., 2010), adult (Meyer et al., 2007) and non-British (Holtmann et al., 2009) populations relying on the HCL. In our study, the risk-taking item loaded onto the active-elated rather than the irritability/risk-taking factor. Results from previous studies are mixed with regard to this item, with some showing it loads on to the irritable/risk-taking subscale while others find it maps onto the active-elated dimension (Brand et al., 2011, Forty et al., 2010).

Higher mean scores on the overall HCL-16 and the active-elated but not the irritable/risk-taking subscales were observed among those with a family history of BD compared to those without such a history. These results concur with the research which shows that family history of BD is a significant risk factor for this disorder and the experience of hypomanic symptoms (Smoller and Finn, 2003, Youngstrom et al., 2009). Overall the results concerning the structure of the HCL-16 suggest it maybe a valid hypomanic instrument for youth in the community.

### 4.2 Associations between hypomania, psychological and psychopathological dimensions

The current study's second objective was to assess the construct validity of the HCL-16 by examining the degree to which HCL-16 scores were correlated with psychopathological (e.g. paranoia) and psychological (e.g. personality) dimensions known to be associated with clinical levels of hypomania and BD. The findings from this study showed that hypomanic symptoms were significantly positively correlated with the majority of the psychopathological dimensions tested with a few exceptions. The associations between hypomania and psychotic-like experiences have not been explored in previous studies using community adolescent samples but results from the current investigation could be viewed as mirroring the significant proportion of people with BD who also experience psychotic features (Dunayevich and Keck, 2000). These findings may highlight a particularly vulnerable group given that the presentation of hypomanic and psychotic symptoms at a clinical level would result in diagnosis of a manic rather than hypomanic episode (APA, 2013).

The correlations between hypomania, internalizing and externalizing problems found in this investigation parallels results of previous studies which have shown high rates of anxiety disorders and ADHD among people with BD (Merikangas et al., 2011). The results of the current study indicate that the psychopathological dimensions that are associated with BD are also linked to hypomanic symptoms in youth, which offers indirect evidence that the HCL-16 has good construct validity.

The relationship between the five main personality dimensions and hypomanic symptoms has not been investigated in previous studies using British nonclinical adolescent populations. The novel results in the current study showed that hypomania was significantly associated with extraversion and openness to experience which is found in other studies using adult and clinical populations (Tackett et al., 2008). Some studies also report that BD is positively associated with neuroticism (Tackett et al., 2008), whereas others show a negative correlation with agreeableness (Barnett et al., 2011). These results further support the validity of using the HCL-16 for assessing hypomanic symptoms among adolescents, and that such symptoms may be linked to extraversion and openness to experience at this developmental stage.

Sleep disturbance is one of the hallmarks of BD (Harvey, 2008), although the investigation of this relationship has been ignored in many studies using community youth samples. The results of this study showed a significant relationship between sleep problems and hypomanic symptoms in a community sample of adolescents. The sleep disturbance measure used in this study covered a range of problems, including those characteristic of hypomania or mania (e.g. reduced need for sleep) and depression (eg. difficulties falling and staying asleep) (APA, 2013). These results provide further support of the validity of the HCL-16 in measuring hypomanic symptoms and show that the relationship between sleep problems and hypomania may already be evident in adolescence.

### 4.3 Comparison between the ‘bright’ and ‘dark’ sides of hypomania on psychological and psychopathological dimensions

This study's third objective was to compare the two hypomania dimensions on psychological and psychopathological correlates. The findings of this study showed that the ‘dark’ side of hypomania showed stronger links with externalizing (e.g. conduct problems) and internalizing (e.g. depression) problems, as well as psychotic-like experiences (e.g. paranoia) relative to the ‘bright’ side. These findings are consistent with a previous study of university students which reported that the ‘dark’ side of hypomania was characterized by symptoms of depression and more perceived stress compared to the ‘bright’ side (Brand et al., 2011).

### 4.4 Identification of group at ‘high-risk’ for BD

The final objective of this investigation was to explore whether the HCL-16 could be used to identify a group of adolescents that show signs of vulnerability for BD. Currently, identification of young people (particularly in community samples) at risk of developing BD involves screening for the presence of hypomanic symptoms (Waugh et al., 2014). Another screening method focuses on other elements of the diagnostic criteria: impact on functioning and symptom duration (Holtmann et al., 2009). When both these approaches are combined a total of 9% of the current sample were identified as being at ‘high-risk’ for hypomania and therefore BD to the extent that they reported the presence of 8 or more hypomanic symptoms (HCL-16 cut-off for hypomania) during ‘highs’ that lasted at least 2 days, which were associated with negative consequences. As expected, the ‘high-risk’ group experienced more psychopathological symptoms relative to the non-hypomanic group, particularly depression, anxiety sensitivity and psychotic-like experiences. These associations provide support that this approach for identifying a ‘high-risk’ group maybe valid, but further research is needed using a longitudinal study to confirm the outcome of the individuals in this sample. In the present study the assessment of sensitivity and specificity of the HCL-16 was not possible since the participants did not receive a clinical assessment for BD.

There is some debate surrounding the possible shorter episode duration in BD (Angst et al., 2003), thus ‘high’ phases of 2 days or more was the focus in this study. But the DSM-5 diagnostic criteria for hypomania requires symptom duration of at least 4 days. In comparing the profile of those in the high-risk group with shorter (2–3 days) to those with longer phase durations (4 days or more) no significant differences were observed in the current study. The lack of significant differences between shorter and longer high phases concurs with the findings of previous studies (Hafeman et al., 2013, Stringaris et al., 2010), which support studying briefer periods of hypomania, especially among youth, to identify those at risk for BD and/or in need of clinical attention.

### 4.5 Clinical implications

Our conclusions are tempered by the fact that our study was cross-sectional, with no follow-up of individuals. Nevertheless, research such as ours, on the characterization of adolescent hypomania, will feed into the development of more effective targeting of prevention and intervention efforts. The findings of this study are clinically valuable since they suggest that using the HCL-16 may help practitioners identify young people vulnerable to developing BD. Second, greater understanding of the hypomanic dimensions and their correlates may provide some explanations surrounding delayed diagnosis of BD. The results from this study show that the active-elated subscale is associated with less psychopathology relative to the irritable/risk-taking dimension. These findings suggest that the active-elated dimension may be linked to fewer problematic issues (eg. better life satisfaction) which means that individuals scoring highly on this dimension are less likely to seek help relative to those with higher scores on the irritable/risk-taking subscale. For this reason it is important that practitioners are aware of the characterization of the active-elated dimension, as reported in this study, to help them determine ‘high-risk’ individuals who are experiencing limited negative consequences and therefore harder to identify.

### 4.6 Methodological strengths and limitations

There are several strengths to the current investigation including the use of a representative community sample of adolescents, which provides the opportunity to investigate the characterization of hypomania likely to be prior to full-blown illness onset and treatment. Similar studies employing clinical samples are limited by a number of possible confounders related to the consequences of the illness. But several weaknesses need to be considered when interpreting the findings from the current investigation.

It was a limitation that the study did not have caregiver or parent reports available in addition to self-report. Research shows that caregiver or parent report concerning BD shows the greatest validity relative to youth and teacher report (Youngstrom et al., 2009). Although the agreement between parent and youth report of BD is significantly higher than usual cross-informant agreement (Youngstrom et al., 2009). Previous research also shows that the HCL has good sensitivity and specificity for identifying BD (Forty et al., 2010). In the present study the hypersexuality/flirtatious item from the HCL-16 was removed for concerns of offending this longitudinal sample, which is unfortunate given that this symptom can help distinguish pediatric BD from other conditions, such as ADHD (Adelson et al., 2013). Future studies replicating the findings here should aim to use the full version of the HCL-16.

Although the sample size was large and consisted of adolescents from across the UK the participants were one half of a twin pair and mainly Caucasian, which may limit the generalizability of this study's findings. Research supports the assumption that self-reported psychiatric symptoms by twins are comparable to singletons (Kendler et al., 1995). Future studies should use ethnically diverse singleton samples to confirm the patterns observed in the current study are relevant to other groups. The exploration of the genetic and environmental architecture of hypomania in this UK adolescent sample was outside the scope of the current study but should be the focus of future investigations, especially since the majority of the existing literature focuses on adult and/or clinical samples (McGuffin et al., 2003).

Substance use may have affected the HCL-16 scores given their significant association in the present study. There are several issues to consider when interpreting the associations between hypomanic symptoms and the other dimensions. For instance, given the large sample size, the correlation coefficients should be the focus here rather than the significance level. Also there was some variation in the index periods. For example, participants reported on their experience of hypomanic symptoms during self-identified ‘high’ periods whereas they rated other measures using specific index periods (eg. within the last month for cognitive disorganization).

The internal consistency of the HCL-16 and its subscales in the present sample reached acceptable levels, except for the irritable/risk-taking scale. The levels of endorsement for the items from the irritable/risk-taking subscale were low (Table 2) and there were fewer items forming this subscale in comparison to other studies that reported higher levels of internal consistency (Forty et al., 2010). It should be noted that previous studies using the HCL-16 have relied on adult clinical samples (Forty et al., 2010) rather than a community adolescent cohort used here, which may explain such discrepancies. Clearly the findings of the present investigation warrant replication to address these issues.

To summarise, this is one of the few studies to examine the profile of hypomania in a large British adolescent community sample. The results showed that hypomania was significantly related to various dimensions of psychopathology, sleep problems and several personality constructs which mirror relationships observed in adult bipolar samples. These findings support the validity of the HCL-16 and, with further research, may represent a possible tool to identify adolescents at risk of developing BD.

## Figures and Tables

**Table 1:** Sample characteristics and key frequencies.

Characteristic	N=1440 N (%)
Female	830 (58%)
Age (mean, SD)	17.05 years (0.88)
White ethnicity	1331 (92%)
Family history of BD	82 (6%)
Hypomania score (mean, SD)	7.12 (2.60)
HCL−16 score of 8 or more (cut-off for hypomania)	673 (47%)
Any type of negative impact or reaction to periods of high mood^a^	535 (37%)
Highs^a^ with a duration of 2 days or more	471 (33%)
Score above HCL−16 cut-off with ‘highs’^a^ lasting at least 2 days and any type of negative impact or reaction	124 (9%)

a Periods of high mood were operationalized in the HCL-16 as phases when the individual experienced increases in their energy, activity and mood.

**Table 2:** Principal component analysis and item endorsement for the HCL-16 in a British adolescent sample (N=1440).

Item	Component 1	Component 2	Item endorsement^a^
	^‘^Active-elated’	‘Irritable/ risk-taking’	
Eigenvalue	2.72	1.77	−
Variance explained (%)	18.16	11.81	−
1. I need less sleep	0.39	0.11	0.41
2. I enjoy work more	0.53	−0.19	0.74
3. I want to travel more/I do travel more	0.43	0.16	0.63
4. I spend more money/ I spend too much money	0.26	0.40	0.48
5. I take more risks in my daily life (in my work or at school and/or other activities)	0.51	0.31	0.53
6. I am more physically active (e.g sports etc.)	0.54	−0.16	0.71
7. I am less shy or inhibited	0.60	−0.03	0.78
8. I wear more colourful and more extravagant clothes/makeup	0.27	0.34	0.26
9. I think faster	0.55	0.07	0.59
10. I make more jokes or puns when I am talking	0.52	0.13	0.76
11. I get into more quarrels	0.01	0.47	0.11
12. My mood is higher, more optimistic	0.63	−0.08	0.87
13. I smoke more cigarettes	−0.18	0.66	0.05
14. I drink more alcohol	0.06	0.63	0.18
15. I take more drugs (e.g. sedatives, anti-anxiety pills, stimulants etc.)	−0.06	0.53	0.02

a Item mean with a higher score showing a greater frequency of endorsement (range 0–1).

**Table 3:** Correlations between hypomania, psychological and psychopathological dimensions.

Dimension	HCL-16 total score^a^	Bright side of hypomania^a^	Dark side of hypomania^a^	Steiger's z-test^b^
		(active-elated)	(Irritable/ risk-taking)	
***Internalizing problems***				
Depressive symptoms	0.23***	0.13***	0.27***	**z=5.18, p≤0.002**
Anxiety sensitivity	0.25***	0.19***	0.25***	z =2.42, p=0.015
*Psychotic-like experiences*				
SPEQ Paranoia	0.25***	0.17***	0.27***	**z =3.85, p≤0.002**
SPEQ Hallucinations	0.29***	0.21***	0.27***	z =2.07, p=0.039
SPEQ Cognitive disorganization	0.20***	0.08***	0.26***	**z =6.35, p≤0.002**
				
SPEQ Grandiosity	0.26***	0.22***	0.23***	z = 0.26, p=0.797
SPEQ Anhedonia	0.22***	0.22***	0.16***	z =2.14, p=0.032
SPEQ Negative symptoms^c^	−0.01	−0.06	0.06	**z =4.07, p≤ 0.002**
				
Psychotic-Like Symptoms (PLIKS-Q)	0.23***	0.15***	0.22***	z =2.68, p=0.007
***Exploratory analyses***^d^				
SDQ Emotional problems	0.12***	0.05	0.16***	**z =3.79, p≤0.002**
SDQ Hyperactivity/inattention	0.15***	0.05	0.19***	**z =5.30, p≤0.002**
				
SDQ Conduct problems	0.17***	0.07	0.20***	**z =4.85, p≤0.002**
				
SDQ Peer problems	0.05	−0.01	0.12***	**z =4.52, p≤0.002**
				
SDQ Prosocial behaviour	0.06	0.09***	0.01	z =2.77, p=0.005
***Personality dimensions***				
Extraversion	0.18***	0.16***	0.13	z=0.81, p=0.417
Neuroticism	0.03	−0.02	0.09	z =2.50, p=0.012
Openness to experience	0.14***	0.13***	0.10	z=0.78, p=0.433
Agreeableness	0.01	0.03	0.003	z =0.53, p=0.594
Conscientiousness	0.02	0.08	−0.01	z =1.95, p=0.05
***Other dimensions***				
Sleep problems	0.20***	0.15***	0.21***	z =1.91, p=0.056
				
Life satisfaction	−0.09***	−0.01	−0.15***	**z =−4.99, p≤0.002**
				

Abbreviations: HCL-16, Hypomania Checklist 16; SPEQ, Specific Psychotic Experiences Questionnaire; PLIKS-Q, Psychotic-Like Symptoms Questionnaire; SDQ, Strengths and Difficulties Questionnaire

*** p≤0.002 (Bonferroni corrected significance level)

a Sample size ranged from 560 to 1438

b Significant differences (set at p≤0.002) between correlations with each dimension and the 2 hypomania sub-scales

c Parent rated, all other scales are based on self-report

d Instruments completed at a different time point to the HCL-16 (exploratory analysis)

**Table 4:** Mean differences between the ‘high-risk’ for hypomania and comparison groups.

**Dimension**	**‘High-risk’ group (N=124)**^a^**Mean (SD) or N (%)**	**Comparison group (N=1316)**^b^**Mean (SD) or N (%)**	**Statistic**^c^	**Cohen's d**^d^
Age	16.99 (0.84)	17.05 (0.88)	t(1438)=0.81, p>0.002	0.07
White ethnic origin	115 (93%)	1216 (92%)	χ^2^(1)=0.001, p>0.002	
Socioeconomic status composite score	0.08 (1.09)	0.02 (0.98)	t(645)=0.47, p>0.002	0.06
Family history of bipolar disorder	7 (6%)	75 (6%)	χ^2^(1)=0.00, p>0.002	
***Internalizing problems***				
Depressive symptoms	6.53 (6.18)	4.17 (5.52)	t(148)=4.15, p≤0.002	**0.40**
Anxiety sensitivity	10.39 (6.18)	7.97 (6.29)	t(1437)=4.09, p≤0.002	**0.39**
***Psychotic-like experiences***				
SPEQ Paranoia	20.45 (14.96)	14.30 (13.66)	t(1435)=4.74, p≤0.002	**0.43**
SPEQ Hallucinations	9.76 (8.30)	6.51 (7.47)	t(1437)=4.58, p≤0.002	**0.41**
SPEQ Cognitive disorganization	5.26 (2.75)	4.42 (3.12)	t(154)=3.20, p≤0.002	**0.29**
SPEQ Grandiosity	6.69 (4.96)	4.57 (4.73)	t(1436)=4.74, p≤0.002	**0.44**
SPEQ Anhedonia	35.73 (6.74)	32.71 (8.15)	t(159)=4.68, p≤0.002	**0.40**
SPEQ Negative symptoms e	3.66 (4.46)	3.73 (4.77)	t(1423)=0.15, p>0.002	0.02
Psychotic-Like Symptoms (PLIKS-Q)	2.27 (2.63)	1.25 (2.20)	t(138)=4.17, p≤0.002	**0.42**
**Exploratory analyses**^f^				
SDQ Emotional problems	3.78 (2.36)	3.10 (2.45)	t(1434)=1.92, p>0.002	**0.28**
SDQ Hyperactivity/inattention	4.19 (2.13)	3.84 (2.54)	t(158)=1.75, p>0.002	0.15
SDQ Conduct problems	2.18 (1.54)	1.80 (1.61)	t(1434)=2.51, p>0.002	**0.24**
SDQ Peer problems	2.16 (1.85)	1.84 (1.77)	t(1434)=1.51, p.0.002	0.18
SDQ Prosocial behaviour	6.88 (2.20)	7.05 (2.07)	t(1433)=0.86, p>0.002	0.08
*Personality dimensions*				
Extraversion	3.61 (0.71)	3.59 (0.68)	t(566)=0.22, p>0.002	0.03
Neuroticism	2.94 (0.65)	2.65 (0.71)	t(567)=2.65, p>0.002	**0.43**
Openness to experience	3.83 (0.57)	3.57 (0.62)	t(565)=2.78, p>0.002	**0.44**
Agreeableness	3.65 (0.61)	3.65 (0.65)	t(564)=0.04, p>0.002	0.00
Conscientiousness	3.68 (0.62)	3.74 (0.66)	t(563)=0.60, p>0.002	0.09
***Other dimensions***				
Sleep problems	6.24 (2.68)	5.99 (3.02)	t(1106)=0.82, p>0.002	0.09
Life satisfaction	5.18 (1.13)	5.49 (1.23)	t(1435)=2.64, p>0.002	**0.26**

Abbreviations: HCL-16, Hypomania Checklist 16; SD, standard deviation; SPEQ, Specific Psychotic Experiences Questionnaire; PLIKS-Q, Psychotic-Like Symptoms Questionnaire, SDQ, Strengths and Difficulties Questionnaire.

a HCL-16 score of 8 or more with ‘highs’lasting at least 2 days with negative consequences

b Please note that there are differences in the sample sizes due to missing data

c Significance was set at p≤0.002 after Bonferonni correction for multiple testing was applied

d Assessing significant differences between the groups focuses on the effect size calculated using Cohen's d, where 0.2 is considered small, 0.5 medium and 0.8 large effects

e Parent-rated, all other scales are based on self-report

f Dimensions measured prior to the HCL-16 thus associations undertaken in exploratory capacity.
