# Supplementary material for: Characterization and structure of hypomania in a British nonclinical adolescent sample
Source: J Affect Disord. 2017 Jan 1;207:228–35. doi: 10.1016/j.jad.2016.08.033 (PMC5113133; doi:10.1016/j.jad.2016.08.033)
Supplement: Supplementary file 1 — Supplementary Material [file mmc1.docx]

***Supplementary Table 1***. Mean differences between those brief (2-3days) and longer (4 or more days) ‘high’ phases among the ‘high-risk’ group.

| **Dimension** | **‘High-risk’ group with symptom duration of least 4 day (N=40)^a b^ Mean (SD) or N (%)** | **‘High-risk’ group with symptom duration of 2-3 days (N=84)^a b^**  **Mean (SD) or N (%)** | **Statistic^c^** | **Cohen’s d ^d^** |
| --- | --- | --- | --- | --- |
| Age | 17.03 (0.83) | 16.98 (0.86) | t(122)=0.32, p>0.002 | 0.05 |
| White ethnic origin | 77 (92%) | 38 (95%) | χ^2^(1)=0.45, p>0.002 |  |
| SES | -0.11 (1.19) | 0.20 (1.02) | t(50)=1.00, p>0.002 | **0.28** |
| Family history of bipolar disorder | 2 (5%) | 5 (6%) | χ^2^(1)=0.07, p>0.002 |  |
| ***Internalizing problems*** |  |  |  |  |
| Depressive symptoms | 5.50 (5.54) | 7.02 (6.43) | t(122)=1.29, p>0.002 | **0.25** |
| Anxiety sensitivity | 9.82 (6.28) | 10.65 (6.15) | t(122)=0.70, p>0.002 | 0.13 |
| ***Psychotic-like experiences*** |  |  |  |  |
| SPEQ Paranoia | 20.39 (14.67) | 20.48 (15.19) | t(121)=0.03, p>0.002 | 0.01 |
| SPEQ Hallucinations | 10.88 (9.76) | 9.23 (7.52) | t(122)=1.03, p>0.002 | 0.19 |
| SPEQ Cognitive disorganization | 4.88 (2.61) | 5.44 (2.81) | t(122)=1.07, p>0.002 | **0.21** |
| SPEQ Grandiosity | 6.83 (4.53) | 6.63 (5.17) | t(121)=0.20, p>0.002 | 0.03 |
| SPEQ Anhedonia | 35.45 (7.38) | 35.86 (6.46) | t(122)=0.32, p>0.002 | 0.06 |
| SPEQ Negative symptoms ^e^ | 3.55 (4.07) | 3.88 (5.22) | t(118)=0.37, p>0.002 | 0.07 |
| Psychotic-Like Symptoms (PLIKS-Q) | 2.54 (2.98) | 2.14 (2.46) | t(121)=0.77, p>0.002 | 0.15 |
| ***Exploratory analyses*** *^f^* |  |  |  |  |
| SDQ Emotional problems | 3.21 (2.37) | 4.05 (2.31) | t(122)=1.86, p>0.002 | **0.36** |
| SDQ Hyperactivity/inattention | 4.35 (2.18) | 4.12 (2.11) | t(122)=0.56, p>0.002 | 0.11 |
| SDQ Conduct problems | 1.93 (1.37) | 2.30 (1.60) | t(122)=1.29, p>0.002 | **0.20** |
| SDQ Peer problems | 2.18 (1.72) | 2.16 (1.92) | t(122)=0.06, p.0.002 | 0.01 |
| SDQ Prosocial behaviour | 7.00 (2.18) | 6.82 (2.22) | t(122)=0.42, p>0.002 | 0.08 |
| *Personality dimensions* |  |  |  |  |
| Extraversion | 3.76 (0.64) | 3.54 (0.74) | t(45)=1.02, p>0.002 | **0.32** |
| Neuroticism | 2.98 (0.68) | 2.85 (0.58) | t(45)=0.63, p>0.002 | **0.21** |
| Openness to experience | 3.82 (0.51) | 3.83 (0.69) | t(45)=0.06, p>0.002 | 0.02 |
| Agreeableness | 3.64 (0.50) | 3.65 (0.67) | t(45)=0.09, p>0.002 | 0.02 |
| Conscientiousness | 3.63 (0.52) | 3.72 (0.68) | t(45)=0.47, p>0.002 | 0.15 |
| *Other dimensions* |  |  |  |  |
| Sleep problems | 6.17 (2.45) | 6.28 (2.80) | t(100)=0.19, p>0.002 | 0.04 |
| Life satisfaction | 5.09 (1.18) | 5.38 (1.02) | t(122)=1.34, p>0.002 | **0.26** |

^a^ HCL-16 score of 8 or more with negative consequences

^b^ Please note that there are differences in the sample sizes due to missing data

^c^ Significance was set at p≤0.002 after Bonferonni correction for multiple testing was applied

^d^ Assessing significant differences between the groups focuses on the effect size calculated using Cohen’s d, where 0.2 is considered small, 0.5 medium and 0.8 large effects

^e^ Parent rated, all other scales are based on self report

^f^ Dimensions measured prior to the HCL-16 thus associations undertaken in exploratory capacity

Abbreviations: HCL-16, Hypomania Checklist 16; SD, standard deviation; SPEQ, Specific Psychotic Experiences Questionnaire; PLIKS-Q, Psychotic-Like Symptoms Questionnaire, SDQ, Strengths and Difficulties Questionnaire.
